# Translation, adaption and validation of the total nasal symptom score (TNSS) for Lithuanian population

**DOI:** 10.1186/s12955-020-01659-8

**Published:** 2021-02-11

**Authors:** Laura Tamasauskiene, Edita Gasiuniene, Brigita Sitkauskiene

**Affiliations:** Department of Immunology and Allergology, Lithuanian University of Health Sciences, Eiveniu str. 2, Kaunas, Lithuania

**Keywords:** Rhinitis, Allergic, TNSS, Nasal symptoms

## Abstract

**Background:**

Allergic rhinitis is one of the most prevalent allergic diseases worldwide which diagnosis is based on typical clinical signs and positive results of allergic tests. Selection and evaluation of treatment is based mainly on subjective symptoms. Objective measurement of patients’ complaints is necessary for proper documentation and follow-up. There are no short simple validated questionnaire assessing nasal symptoms in patients with allergic rhinitis in Lithuania. Total nasal symptoms score (TNSS) is a brief questionnaire which evaluate the severity of main symptoms of allergic rhinitis widely used in different countries. Our aim was to translate the TNSS in the Lithuanian language and to validate it.

**Methods:**

Prospective cross-cultural adaption and validation study was performed. Linguistic validation of TNSS was performed and validity and reliability were assessed. Patients with chronic allergic and non-allergic rhinitis and healthy individuals were included in this study. Patients had to complete translated version of TNSS. Patients with allergic rhinitis additionally were asked to fill Rhinoconjunctivitis Quality of Life Questionnaire (RQLQ).

**Results:**

Seventy-six individuals were involved into the study: 16 with non-allergic rhinitis (NAR) (21.1%), 49 with allergic rhinitis (AR) (64.5%) and 11 healthy individuals (14.5%). Cronbach’s α was 0.87. TNSS score was significantly higher in patients with NAR and AR compared with healthy individuals (3.56 ± 2.28 vs. 4.28 ± 2.46 vs. 0.27 ± 0.91). Positive significant correlation was found between TNSS score and RQLQ score (rs = 0.77, *p* < 0.01).

**Conclusions:**

The Lithuanian version of the TNSS proved to be a valid instrument for assessing nasal symptoms in patients with allergic rhinitis.

## Introduction

Chronic rhinitis affects about 30% population worldwide [1]. Allergic rhinitis is one of the most common phenotypes of this disease and one of the most prevalent allergic diseases worldwide [1–4]. Diagnosis of allergic rhinitis is based on typical clinical signs (rhinorrhea, nasal obstruction or blockage, nasal itching, sneezing, and postnasal drip) and positive results of allergic tests [4]. Treatment of allergic rhinitis is chosen according to the severity of the disease, which is determined by clinical symptoms [4]. Evaluation of treatment efficacy is also based on subjective symptoms. Objective measurement of patients’ complaints is necessary for proper documentation and follow-up. That is why it is very important to have simple standardized tool for evaluation of symptoms of allergic rhinitis in daily clinical practice. Moreover, cross-cultural adapted and validated questionnaire is necessary for international comparison of studies.

In Lithuania there are validated and adapted two questionnaires which assess nasal symptoms—sino-nasal outcome test (SNOT)-22 [5] and Rhinoconjunctivitis Quality of Life Questionnaire (RQLQ) [6]. However, to answer these questionnaires takes quite long time and they are rarely used in routine medical practice. Total nasal symptoms score (TNSS) is a brief questionnaire which evaluate the severity of main symptoms of allergic rhinitis [7]. It consists of three questions which assess nasal obstruction, itching/sneezing and secretion/runny nose. Each question can be answered using a 4-point scale from “0” (no symptoms) up to “3” (severe symptoms). TNSS has been already validated and adapted in many European countries such as Netherlands, Ireland, United Kingdom, France, Germany, Greece, Italy, Sweden and Spain, and in Canada and United States of America. Our aim was to translate the TNSS in the Lithuanian language and to validate it.

## Methods

### Study design

Prospective cross-cultural adaption and validation study was performed in 2019 at the Department of Immunology and Allergology, Hospital of Lithuanian University of Health Sciences. Permission was received from Mapi Research Trust.

### Linguistic validation

The linguistic validation of the TNSS from English to Lithuanian consisted of three steps: a forward translation, a backward translation and cognitive interviews. Two independent, bilingual Lithuanian-native speakers with medical background (allergist and clinical immunologist) translated the original English version into Lithuanian. A consensus version was developed by discussion and revision of the translated versions by the authors. Backward translation of the consensus version was performed independently by one English native speaker without medical background, who was familiar with cultural and linguistic nuances of the original and translated language. Adequacy of the translated version was proven by comparison of the original with the backward-translated versions. (Table 1). Then patient testing was performed. The individual interviews were performed during which the interviewer inquired whether the participant had any difficulty in understanding the questionnaire and checked the participant’s interpretation of all items. Finally, proofreading was performed.

**Table 1:** Original version of the TNSS with adapted translations in Lithuanian language (italic) (sample copy, do not use without permission)

1. Nasal obstruction *Nosies užgulimas*	
No symptoms *Nebuvo*	0
Mild—awareness but not troubled *Lengvas – buvo, bet nevargino*	1
Moderate—troublesome but not interfering with normal daily activities or sleep Vidutinio sunkumo – vargino, bet netrukdė įprastai kasdienei veiklai ar miegui	2
Severe—interfering with normal daily activities or sleep *Sunkus – trukdė įprastai kasdienei veiklai ar miegui*	3
2. Itching/Sneezing *Niežulys/čiaudulys*	
No symptoms *Nebuvo*	0
Mild—awareness but not troubled *Lengvas – buvo, bet nevargino*	1
Moderate—troublesome but not interfering with normal daily activities or sleep *Vidutinio sunkumo – vargino, bet netrukdė įprastai kasdienei veiklai ar miegui*	2
Severe—interfering with normal daily activities or sleep *Sunkus – trukdė įprastai kasdienei veiklai ar miegui*	3
3. Secretion/runny nose *Nosies sekreto varvėjimas*	
No symptoms *Nebuvo*	0
Mild—awareness but not troubled *Lengvas – buvo, bet nevargino*	1
Moderate—troublesome but not interfering with normal daily activities or sleep *Vidutinio sunkumo – vargino, bet netrukdė įprastai kasdienei veiklai ar miegui*	2
Severe—interfering with normal daily activities or sleep *Sunkus – trukdė įprastai kasdienei veiklai ar miegui*	3
Total score *Bendras balas*	

### Reliability

The reliability of a test was defined by its internal consistency (Cronbach’s α value). Internal consistency was considered to be fair (0.7 ≤ α ≤ 0.79), good (0.8 ≤ α ≤ 0.89) or excellent (α ≥ 0.9). Corrected item-total and inter-item correlations were determined by Spearman correlation (0.2 < rsp ≤ 0.5: low correlation, 0.5 < rsp ≤ 0.8: good correlation, 0.8 < rsp ≤ 1.0: excellent correlation).

### Validity

The validity consists of the construct and the discriminant validity. Spearman’s correlation between the TNSS sum score and separate TNSS questionnaires scores and the RQLQ sum score and separate RQLQ part assessing nasal symptoms score were determined to describe the construct validity. Comparison of TNSS sum score and separate TNSS questionnaires scores between healthy individuals and patients with AR and NAR using Kruskal–Wallis test was performed for determination of the discriminant validity.

### Study population

Patients with chronic allergic and non-allergic rhinitis were included in this study. Allergy was assessed using skin prick test and/or measurement of allergen specific immunoglobulin E in blood. All patients with allergic rhinitis had hypersensitivity to perennial allergens. Chronic (perennial) allergic and non-allergic rhinitis were diagnosed according to Allergic rhinitis and its impact on asthma (ARIA) and European Academy of Allergy and Clinical Immunology (EAACI) recommendations [1, 4]. Healthy volunteers without nasal diseases were involved in control group. All participants were asked to complete the translated TNSS questionnaire during outpatient consultation. Patients with allergic rhinitis additionally were asked to fill RQLQ.

The study was approved by the Kaunas Regional Bioethics Committee (No. BE-2-28). Subjects gave their informed consent.

### Statistical analysis

Statistical analysis was performed using statistical program IBM® SPSS® Statistics 24. Kruskal–Wallis test was used for comparison of independent non-parametric variables between several groups. Correlation was assessed using Spearman’s coefficient. The reliability was calculated using Cronbach’s α value. A *P* value of < 0.05 was considered statistically significant.

## Results

### Study population

Seventy-six individuals were involved into the study: 16 with non-allergic rhinitis (NAR) (21.1%), 49 with allergic rhinitis (AR) (64.5%) and 11 healthy individuals (14.5%). Characteristics of the studied population are shown in Table 2.

**Table 2:** Characteristics of studies population

	NAR	AR	Healthy individuals
Female/male, n	8/8	28/21	8/3
Age, years, mean ± SD	37.13 ± 15.70	31.08 ± 10.99	34.50 ± 13.40
Duration of nasal symptoms, years, mean	7	13	0
Individuals who use treatment for rhinitis, n (%)	4 (25%)	19 (38.8%)	0 (0%)

### Reliability

Cronbach’s α was 0.87. It shows high internal consistency of the translated TNSS. Intraclass correlation coefficients confirmed the high internal consistency at each time point (ICC 0.87 (95% CI 0.80; 0.91). The inter-item and item-total correlations are illustrated in Table 3. All correlations were good or excellent and statistically significant.

**Table 3:** Inter-item and item-total correlations of the TNSS (Spearman’s coefficient)

	Nasal obstruction	Itching/sneezing	Secretion/runny nose
Itching/sneezing	0.64**		
Secretion/runny nose	0.78**	0.67**	
TNSS	0.91**	0.85**	0.92**

**p < 0.01

### Validity

TNSS score was significantly higher in patients with NAR and AR compared with healthy individuals (Table 4). Separate TNSS questions scores were also significantly higher in patients with NAR and AR than in control group except for itching/sneezing (this score was significantly higher only in AR patients compared with healthy individuals) (Table 4).

**Table 4:** TNSS score in studied groups

	NAR	AR	Healthy individuals
TNSS	3.56 ± 2.28**	4.28 ± 2.46**	0.27 ± 0.91
Nasal obstruction	1.70 ± 0.95**	1.51 ± 0.98**	0.09 ± 0.30
Itching/sneezing	0.81 ± 0.75	1.47 ± 0.91**	0.09 ± 0.30
Secretion/runny nose	1.13 ± 1.02**	1.30 ± 0.94**	0.09 ± 0.30

Data are presented as mean ± SD

**p < 0.01 compared with healthy individuals

Positive significant good or excellent correlations between TNSS score and separate TNSS questions score with RQLQ and separate RQLQ domain assessing nasal symptoms were estimated (Table 5).

**Table 5:** Correlation between RQLQ and its domain assessing nasal symptoms and TNSS and separate TNSS questions (Spearman’s coefficient)

	RQLQ	RQLQ domain assessing nasal symptoms
TNSS	0.77**	0.85**
Nasal obstruction	0.70**	0.76**
Itching/sneezing	0.55**	0.64**
Secretion/runny nose	0.70**	0.79**

**p < 0.01

## Discussion

This study showed that TNSS is a reliable and valid test for evaluation of nasal symptoms in Lithuanian population. Cronbach’s α was high indicating high internal consistency. The inter-item and item-total correlations were also good or excellent. These parameters showed high reliability of TNSS.

TNSS score was significantly higher in patients with non-allergic rhinitis and allergic rhinits compared with healthy individuals, indicating high discriminant validity. The similar results were also found during the validation of original TNSS [7]. The mean TNSS over 1 year for the control group was 0.7 and for the subjects with allergic rhinitis was 3.7 [7]. Study performed by Badorrek et al. also revealed that TNSS was statistically significantly increased in patients with allergic rhinitis but not in healthy participants [8].

In our study TNSS did not differ between patients with non-allergic rhinitis and allergic rhinitis showing that this tool may be suitable for patients who suffer from nasal symptoms caused by various factors. However, therapeutic indication of TNSS is allergic rhinitis [7, 9].

We found positive significant correlations between TNSS score and RQLQ and separate RQLQ domain assessing nasal symptoms, suggesting high construct validity of TNSS. In agreement with our results, study performed by Garris et al. showed that mean change from baseline in TNSS was strongly correlated with mean change from baseline in RQLQ nasal (r 5 0.69) symptom domain [9].

It is very important to evaluate objectively subjective nasal symptoms before specific treatment (e.g., allergen immunotherapy) and during the follow-up visits. TNSS is widely used in scientific studies including clinical trials (e.g. [10–12]) and in clinical practice by scientists and physicians from different countries. TNSS is recommended in *International Consensus Statement on Allergy and Rhinology: Allergic Rhinitis* as one of the validated symptom severity surveys [13]. TNSS is a short and easily understandable tool; so, it will play a significant role for evaluating nasal symptoms in daily clinical practice in Lithuania. Moreover, we will be able to use this questionnaire in our research and this let us easier to compare our results to studies conducted by others.

## Conclusions

TNSS was successfully translated and validated for Lithuanian population. To sum up, the Lithuanian version of the TNSS proved to be a valid instrument for assessing nasal symptoms in patients with allergic rhinitis.

## Data Availability

Not applicable.

## Abbreviations

TNSS: Total nasal symptoms score
RQLQ: Rhinoconjunctivitis Quality of Life Questionnaire
NAR: Non-allergic rhinitis
AR: Allergic rhinitis
SNOT-22: Sino-nasal outcome test-22
